# Intra-Species Diversity and Panmictic Structure of *Cryptosporidium parvum* Populations in Cattle Farms in Northern Spain

**DOI:** 10.1371/journal.pone.0148811

**Published:** 2016-02-05

**Authors:** Ana Ramo, Joaquín Quílez, Luis Monteagudo, Emilio Del Cacho, Caridad Sánchez-Acedo

**Affiliations:** 1 Department of Animal Pathology, Faculty of Veterinary Sciences, University of Zaragoza, Zaragoza, Spain; 2 Department of Anatomy, Embriology and Genetics, Faculty of Veterinary Sciences, University of Zaragoza, Zaragoza, Spain; Beijing Institute of Microbiology and Epidemiology, CHINA

## Abstract

The intra-herd and intra-host genetic variability of 123 *Cryptosporidium parvum* isolates was investigated using a multilocus fragment typing approach with eleven variable-number tandem-repeat (VNTR) loci and the GP60 gene. Isolates were collected from intensively farmed diarrheic pre-weaned calves originating from 31 dairy farms in three adjoining regions in northern Spain (País Vasco, Cantabria and Asturias). The multilocus tool demonstrated an acceptable typeability, with 104/123 samples amplifying at all twelve loci. The ML2, TP14, GP60 and the previously un-described minisatellite at locus cgd2_3850 were the most discriminatory markers, while others may be dismissed as monomorphic (MSB) or less informative (CP47, ML1 and the novel minisatellites at loci Cgd1_3670 and Cgd6_3940). The 12-satellite typing tool provided a Hunter-Gaston index (HGDI) of 0.987 (95% CI, 0.982–0.992), and differentiated a total of 70 multilocus subtypes (MLTs). The inclusion of only the four most discriminatory markers dramatically reduced the number of MLTs (n: 44) but hardly reduced the HGDI value. A total of 54 MLTs were distinctive for individual farms, indicating that cryptosporidiosis is an endemic condition on most cattle farms. However, a high rate of mixed infections was detected, suggesting frequent meiotic recombination. Namely, multiple MLTs were seen in most farms where several specimens were analyzed (90.5%), with up to 9 MLTs being found on one farm, and individual specimens with mixed populations being reported on 11/29 farms. Bayesian Structure analysis showed that over 35% of isolates had mixed ancestry and analysis of evolutionary descent using the eBURST algorithm detected a high rate (21.4%) of MLTs appearing as singletons, indicating a high degree of genetic divergence. Linkage analysis found evidence of linkage equilibrium and an overall panmictic structure within the *C*. *parvum* population in this discrete geographical area.

## Introduction

*Cryptosporidium parvum* is a prevalent and clinically important pathogen causing gastrointestinal disease in a variety of vertebrate hosts, particularly humans and neonatal domestic ruminants. This protozoan has emerged as a cause of significant economic losses for farmers and a challenging organism for public health professionals [1], but molecular studies have showed that *C*. *parvum* is far from a genetically uniform species, and genetic traits segregating with host range or geographical location have been documented [2–5]. Additionally, the life cycle of *Cryptosporidium* has a sexual phase during which meiotic recombination between genetically distinct lineages can occur, although the relative contribution of recombination to the diversification of the parasite in nature is unknown [6, 7]. In fact, the use of next generation sequencing applications and other tools such as automated fragment analysis have revealed an unexpectedly high intra-isolate genetic heterogeneity, with important implications for our understanding of cryptosporidiosis epidemiology [8–10]. All these data highlight the need of validated tools to unravel the population genetic structure and elucidate the transmission pathways of *C*. *parvum*.

At least 14 subtype families (IIa to IIo) have been identified on the basis of partial sequence analysis of the 60 KDa glycoprotein (GP60) gene. Some families, especially IIc, have so far only been found in humans, but other families, including IIa and IId, are found in both humans and ruminants and are responsible for zoonotic cryptosporidiosis. In particular, IIa is the most frequently reported subtype family in cattle worldwide [11]. However, GP60 subtyping is usually based on conventional Sanger sequencing, which is not effective in detecting the presence of mixed infections, and has mostly been used as a single-locus method, which is unreliable for investigating the population structure of sexually reproducing organisms [7]. A second approach exploits the high variability of short variable-number tandem repeat (VNTR) loci, also known as minisatellites and microsatellites, which are being increasingly used in multilocus studies [11]. Three different and opposing genetic structures have been proposed for *C*. *parvum*, from a panmictic population in which sexual recombination is frequent, to a clonal theory, and an epidemic population structure where there is a background level of frequent sexual recombination with the occasional clonal expansion of a few particular haplotypes [11].

In Spain, the economic significance of *C*. *parvum* is well recognized as one of the most common enteropathogens associated with diarrhea in suckling calves, with prevalence rates over 50% [12–14]. However, the intra-species diversity of *C*. *parvum* in cattle farms has been scarcely explored. GP60 subtyping studies have showed the predominance of the subtype family IIa and in particular the major zoonotic subtype IIaA15G2R1 [14, 15]. Multilocus studies with six VNTR markers conducted in an extensive area covering 14 provinces across northern Spain revealed a high genetic variability, and two host-associated subpopulations showing epidemic clonality were identified among *C*. *parvum* isolates infecting calves and lambs/goat kids, respectively [3, 16]. In the current study, dairy cattle farms with similar husbandry systems in a much more restricted area in northern Spain were sampled to investigate further the intra-herd and intra-host *C*. *parvum* genetic diversity at a smaller geographical scale. Likewise, an expanded panel of markers combining eleven VNTR loci and the GP60 subtype was used, in order to select those more informative and have a more robust examination of the population structure and the role of genetic exchange in this population.

## Materials and Methods

### Ethics Statement

*Cryptosporidium* isolates were obtained from feces collected for diagnostic purposes from calves after the permission of farm owners, with no specific permits being required by the authority for the feces collections. Animal care and use committee approval was not necessary for this study. Directive 2010/63/EU of the European Parliament on the protection of animals used for scientific purposes does not apply to non-experimental clinical veterinary practices.

### *Cryptosporidium* isolates

**Fig 1:**
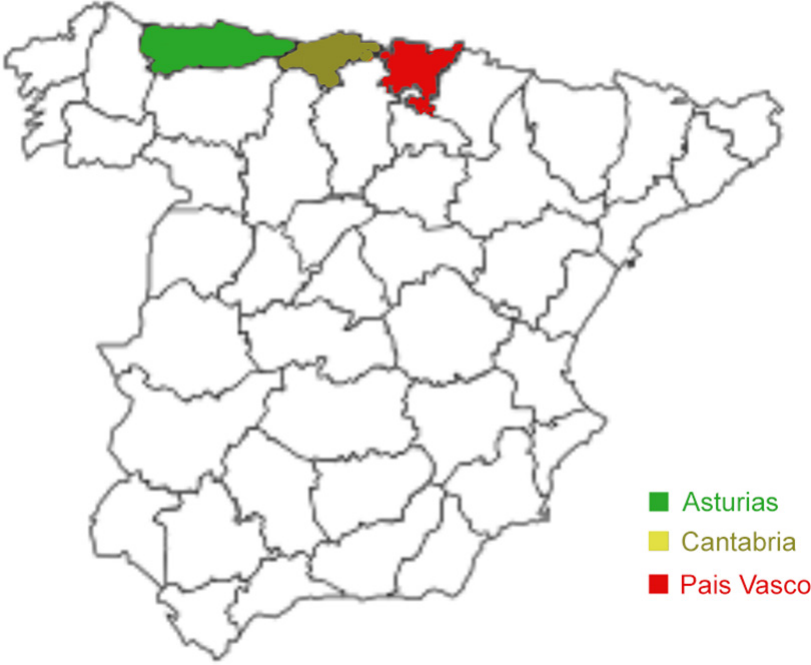
Map of Spain showing the three autonomous regions where cattle farms were sampled.

Genomic DNA samples of *Cryptosporidium parvum* isolates (n: 123) from a previous optimization study of a CE-based tool for identification of *Cryptosporidium* species and subtypes infecting domestic ruminants were used [10]. These isolates had been collected between 2010 and 2013 from naturally infected diarrheic Holstein-Friesian pre-weaned calves originating from 31 dairy cattle farms under intensive farming. Only one specimen from each calf was analyzed. The farms were located in three adjoining autonomous regions (País Vasco, Cantabria and Asturias) covering an area of approximately 23,160 km^2^ in the north of Spain (Fig 1). In 2014, the number of dairy cows in this area was 170,466 animals, which represents 19.4% of the total dairy cattle population in Spain (Ministry of Agriculture, Food and Environment, http://www.magrama.gob.es). *Cryptosporidium* species and *C*. *parvum* GP60 alleles in the previous study were determined based on a fragment size typing approach at the small-subunit (SSU) rRNA and GP60 genes, respectively.

### Multilocus fragment typing

An automated capillary electrophoresis (CE)-based DNA fragment analysis tool combining three fluorochromes was used to type *C*. *parvum* isolates at eleven VNTR markers, including two minisatellites (MSB, MSC6-7) and five microsatellites (ML1, ML2, TP14, 5B12, CP47) previously reported, as well as four previously un-described minisatellite regions at loci cgd2_3850, cgd1_3670, cgd6_5400 and cgd6_3940 of *C*. *parvum*. The four latter markers were identified using the software program SSR Finder (http://www.fresnostate.edu/ssrfinder) on the *C*. *parvum* complete genome (http://cryptodb.org/cryptodb/). Fragments were amplified by using single (MSB, ML2, 5B12, cgd1_3670 and cgd6_3940), heminested (ML1 and cgd6_5400) and nested (TP14, CP47, MSC6-7 and cgd2_3850) PCRs using protocols described previously [16, 17], but new primer sets designed with Primer3 (v. 0.4.0) (http://bioinfo.ut.ee/primer3-0.4.0/primer3/) were used to amplify the novel VNTR regions [18]. In order to allocate alleles with overlapping peaks to a specific locus, the reverse primers used in simple PCRs and the internal reverse primers used in heminested and nested PCRs were 5’ labeled with HEX (4,7,2’,4’,5’,7’-hexachloro-6-carboxyfluorescein), FAM (6-carboxyfluorescein), or TAMRA (6-carboxytetramethylrhodamine), according to the predicted fragment size. The primers used for PCR analysis of all gene targets, the annealing temperatures used, and the sizes of the expected PCR products are listed in Table 1.

**Table 1:** Primers and conditions used for PCR amplification of mini- and microsatellite typing, including four novel VNTR regions identified in the *C*. *parvum* genome in this study using the SSR Finder software.

Locus	GenBank accession no.	Chromosome	Primer	Primer sequence (5’ → 3’)	Repeat motif	Annealing temp (°C)	Fragment size range (bp)	Reference
ML2	AF344880	VI	F	caatgtaagtttacttatgattat	AG	50	180–237	[19]
			R	FAM-cgactataaagatgagagaag				
5B12	AQ449854	II	F	tgacgatgaagatgagggaac	AT	60	134–155	[16]
			R	HEX-caggacagatttaggaggagga				
MSB	XM627997	I	F	gggaggcatagggatga	AGATAG	59	246–324	[20]
			R	TAMRA-cttttgatcgcttcttttcca				
TP14	XM627041	VIII	F1	taatgcccacccatcttctt	CAA	61	279–333	[16, 21]
			R1	tccatctgggtccatttagc				
			F2	ctaacgttcacagccaacagtacc		62		
			R2	FAM-gtacagctcctgttcctgttg				
ML1	G35348	III	F1	catgagctaaaaatggtgg	GAG	55	218–242	[19, 22]
			F2	ctaaaaatggtggagaatattc		50		
			R	HEX-caacaaaatctatatcctc				
CP47	AF384127	VI	F1	gcttagattctgatatggatctat	TAA,TGA/TAG	43	417–479	[17]
			R1	agcttactggtcctgtatcagtt				
			F2	accccagaaggcggaccaaggtt		55		
			R2	FAM-gtatcgtggcgttctgaattatcaa				
MSC6-7	BX538350	VI	F1	attgaacaaacgccgcaaatgtaca	TGATGATGAT(G)GAACC(T)	55	517–570	[17]
			R1	cgattatctcaatattggctgttattgc				
			F2	gctatttgctatcgtctcacataact		55		
			R2	TAMRA-ctactgaatctgatcttgcatcaagt				
cgd2_3850	XM626569	II	F1	attgaagattgcggatgatggggtt	CTGCTC(A)	70	151–205	This study
			R1	tggagcgccaagtgctgaaga				
			F2	atttgctgttgcaactggtg		61		
			R2	TAMRA-gccaagtgctgaagaagagg				
cgd1_3670	XM628179	I	F	ggaaaggcattaatcctcca	TGAGCC	60	235–289	This study
			R	FAM-gaactaggctcgggttcaga				
cgd6_5400	XM627858	VI	F	taatctttgcgtgggacctc	GTAGTG(A)	60	251–312	This study
			R1	gtgacttgaatgacccagga				
			R2	HEX-tggagtttctgagacacaaaga		59		
cgd6_3940	XM627731	VI	F	agtggtcttggagcctgaga	TTGGCA	62	312–342	This study
			R	TAMRA-acttggacaagtgccaggtc				

The CP47 and MSC6-7 loci were amplified by nested PCRs as previously described [17]. All other loci were amplified under the following conditions. In brief, the PCR mixture for single PCRs consisted of 1× PCR buffer, 1.5 mM MgCl_2_, a 200 μM concentration of each deoxynucleoside triphos-phate (dNTP), 1μM (each) forward and reverse primers, 1 U of *Taq* polymerase (Bioron, Germany), and 2 μl of the DNA template in a total reaction volume of 20 μl. Templates were subjected to 40 cycles consisting of 94°C for 30 s, the marker-specific annealing temperature for 30 s, and 72°C for 1 min, with an initial denaturation step at 94°C for 5 min and a final extension at 72°C for 7 min. The PCR mixture for heminested and nested PCRs consisted of 2 μl of DNA template (for primary PCR) or 2 μl of primary PCR product (for secondary PCR), 1× PCR buffer, 2.5 mM MgCl_2_, a 200 μM concentration of each dNTP, a 0.5 μM concentration of each primer, and 1 U of *Taq* polymerase in a total reaction volume of 20 μl. Each PCR mix for heminested and nested reactions was subjected to an initial denaturation at 95°C for 3 min followed by 35 cycles consisting of denaturation at 95°C for 50 s, the marker-specific annealing temperature for 50 s, and extension at 72°C for 1 min, and then a final extension at 72°C for 10 min.

PCR products were first separated by electrophoresis in 1.5% agarose gels and visualized by staining with GelRed nucleid acid gel stain (Biotium, Hayward, CA) to confirm DNA amplification. According to the amplicon intensity, 0.5-to-2 μl samples of the mini- and microsatellite-labeled PCR products for each *C*. *parvum* isolate were mixed and subjected to capillary electrophoresis on a 3500xL Genetic Analyzer and sized automatically using the GeneScan 600 Liz Size Standard (Applied Biosystems, Life Technologies). The data were stored and analyzed with the aid of Gene Mapper software (version 4.1) to determinate fragment sizes. Dye-labeled amplicons were identified in the form of multicolored electropherograms with eleven peaks of fluorescence for each isolate. Each peak corresponded to the length of the PCR product and was allocated to an allele according to the fluorescent label and predicted amplicon size for each marker. The presence of two separate peaks for a specific locus differing by multiples of the repeat unit was assumed to indicate a mixed infection [19]. At least two representative isolates for each allele were amplified using unlabeled primers and the above-mentioned PCR conditions and analyzed by bidirectional sequencing for length confirmation. Size discrepancies between CE and sequencing were found at all VNTR loci and ranged from 1 and 7 bp. Allele nomenclature was based on the fragment size (in base pairs) adjusted after comparison with sequence analysis of these representative isolates. Alleles were translated into numbers for multilocus analyses. Representative sequences generated in this study were deposited in the GenBank database under accession numbers KT806056 to KT806077.

### Multilocus subtype identification

The multilocus subtype (MLT) for each isolate was determined based on the combination of alleles at all eleven VNTR loci and the previously reported GP60 allele, and each MLT was then designated by a number. Only isolates that amplified at all loci were included in the analysis. When two alleles were identified at one locus, the two potential multilocus subtypes were considered. When more than one locus showed several alleles, multilocus subtypes could not be determined and the isolate was excluded from the population genetic analyses. The discriminatory power of each individual marker and the multilocus analysis for typing *C*. *parvum* isolates was assessed by calculating the Hunter-Gaston discriminatory index (HGDI), based on the probability that two unrelated strains sampled from the test population will be placed into different typing groups [23]. The index and 95% confidence intervals were calculated using the online tool V-DICE (Variable Number Tandem Repeat Diversity and Confidence Extractor; http://www.hpa-bioinformatics.org.uk/cgi-bin/DICI/DICI.pl).

### Data analysis

The relationship among the MLTs was estimated using the eBURST algorithm with the Phyloviz software version 1.0 (http://www.phyloviz.net) [24]. The most stringent setting was used, and only MLTs that differ from one another at one locus [single locus variants (SLVs)] were assigned to the same cluster. Clusters of linked SLVs are referred to as clonal complexes. Each cluster is formed around a founder member, which has the highest number of SLVs and is considered to be the common ancestor. Linkage analysis was performed using LIAN v. 3.5 (http://guanine.evolbio.mpg.de/cgi-bin/lian/lian.cgi.pl/query), which tests the null hypothesis of complete panmixia. The LIAN software program calculates the standardized index of association (I_A_^S^), a value that fluctuates randomly around zero in complete panmixia but moves further from zero as linkage disequilibrium increases [25]. The population structure was investigated using STRUCTURE v. 2.3 (http://pritchardlab.stanford.edu/structure.html) [26]. This software uses a Bayesian model based algorithm to identify genetically distinct subgroups on the basis of allelic frequencies. For each *K* value (number of genetic groups), 10,000 burn-in iterations followed by a run of 100,000 Markov chain Monte Carlo repetitions were performed and four repeats of each calculation were done to evaluate variability. The most appropriate number of clusters was determined by comparing values of *K* between 1 and 10. GENETIX software (http://www.genetix.univ-montp2.fr/genetix/genetix.htm) was used to assess the robustness of the sub-structuring, calculating the Wright’s fixation index values (Fst) for the different subpopulations [27].

## Results

### Allelic diversity at VNTR loci

The Table 2 summarizes the identity of alleles and HGDI values at each VNTR locus. A total of 104 *C*. *parvum* isolates from 29 cattle farms were successfully typed at all twelve loci. The number of allelic variants per locus ranged from one to ten. The MSB locus was monomorphic and four other markers showed low variability, with 2–4 alleles each but more than 91% specimens being allocated to a single allele. The latter group included ML1, CP47 and the novel loci cgd1_3670 and cgd6_3940, which displayed a HGDI value lower than 0.2. A higher diversity was seen at the GP60 and the novel Cgd3_3850 loci, which showed each six different alleles, including a predominant allele exhibited by more than 74% of isolates. Only two markers provided an HGDI value higher than 0.6, namely the TP14 and specially the ML2 microsatellites, with the latter exhibiting the highest number of allelic variants (n = 10). Sequencing of isolates selected for fragment length confirmation revealed novel alleles at ML2 (239bp), CP47 (429bp) and MSC6-7 (564bp) loci. The remaining alleles at previously described VNTR markers showed 100% identity to *C*. *parvum* reference sequences deposited in GenBank.

**Table 2:** Adjusted allele sizes and number allocation for each of eleven VNTR loci and the GP60 marker identified by CE in *C*. *parvum* isolates from diarrheic pre-weaned calves.

Locus and adjusted fragment size (bp) (allele no.)^a^	No. of isolates (%) (n = 104)	N° of farms (n = 29)
**ML1 [HGDI = 0.170 (0.083–0.258)]** ^**b**^		
226 (1)	4 (3.8)	3
238 (2)	95 (91.3)	29
241 (4)	1 (0.9)	1
226 + 238	4 (3.8)	1
**TP14 [HGDI = 0.663 (0.645–0.682)]**		
324 (1)	43 (41.3)	17
333 (2)	37 (35.6)	20
342 (3)	19 (18.3)	10
324 + 342	1 (0.9)	1
333 + 342	4 (3.8)	4
**ML2 [HGDI = 0.829 (0.797–0.860)]**		
191 (2)	1 (0.9)	1
193 (9)	7 (6.7)	2
209 (13)	1 (0.9)	1
227 (3)	10 (9.6)	6
229 (4)	10 (9.6)	7
231 (5)	31 (29.8)	17
233 (6)	21 (20.2)	12
235 (7)	13 (12.5)	6
237 (8)	5 (4.8)	2
239 (14)	2 (1.9)	2
193 + 233	1 (0.9)	1
193 + 235	1 (0.9)	1
227 + 233	1 (0.9)	1
**5B12 [HGDI = 0.231 (0.132–0.330)]**		
165 (1)	5 (4.8)	5
167 (2)	4 (3.8)	3
169 (3)	88 (84.6)	29
171 (4)	4 (3.8)	4
167 + 169	2 (1.9)	2
169 + 171	1 (0.9)	1
**MSB [HGDI = 0.000 (0.000–0.059)]**		
322 (3)	104 (100)	29
**CP47 [HGDI = 0.033 (0.000–0.077)]** ^**c**^		
417 (1)	102 (98.1)	29
429 (2)	2 (1.9)	1
**MSC6-7 [HGDI = 0.208 (0.120–0.296)]**		
549 (1)	91 (87.5)	29
564 (2)	9 (8.6)	4
549 + 564	4 (3.8)	4
**Cgd3_3850 [HGDI = 0.347 (0.244–0.450)]**		
151 (1)	5 (4.8)	3
163 (2)	1 (0.9)	1
181 (3)	2 (1.9)	1
193 (4)	84 (80.8)	27
199 (5)	10 (9.6)	4
205 (6)	2 (1.9)	1
**Cgd1_3670 [HGDI = 0.097 (0.025–0.169)]**		
229 (1)	1 (0.9)	1
265 (2)	99 (95.2)	29
271 (3)	1 (0.9)	1
283 (4)	3 (2.9)	3
**Cgd6_5400 [HGDI = 0.211 (0.120–0.303)]**		
277 (1)	91 (87.5)	29
283 (2)	11 (10.6)	4
312 (3)	2 (1.9)	2
**Cgd6_3940 [HGDI = 0.126 (0.048–0.205)]**		
312 (1)	1 (0.9)	1
330 (2)	5 (4.8)	3
336 (3)	98 (94.2)	29
**GP60 [HGDI = 0.412 (0.320–0.505)]**		
333 (1)	1 (0.9)	1
345 (2)	2 (1.9)	1
351 (3)	77 (74)	26
354 (4)	2 (1.9)	1
357 (5)	18 (17.3)	9
363 (6)	1 (0.9)	1
351 + 357	3 (2.9)	2

^a^ Alleles were compared and correlatively numbered according to those identified within *Cryptosporidium* isolates from calves by Quílez et al. [3, 16]

^b^ Hunter-Gaston discriminatory power (discriminatory index [95% confidence interval])

^c^ Alleles at the CP47 locus were identified as IIA29G10 (1) and IIA33G10 (2) by sequencing, according to the nomenclature proposed by Gatei et al. [17]

### Multilocus subtypes

Altogether, 70 multilocus subtypes (MLTs) were identified among 102 *C*. *parvum* specimens, based on the combination of alleles at the twelve VNTR loci (Table 3). A similar number (n: 69) was differentiated when the three less polymorphic markers (MSB, CP47, Cgd1_3670) were excluded in the analysis. Eighteen isolates from ten farms showed a biallelic profile at one locus and were scored each as having two different MLTs. Half of these specimens were from only two farms and exhibited a biallelic profile at any of five loci. Two isolates showed multiples alleles at more than one locus and were excluded from the multilocus analysis. A total of 54 MLTs were distinctive for individual farms, although the presence of multiple MLTs within farms was also common, being reported in 19 out of 21 farms where two or more specimens were collected. The number was related to the number of specimens analyzed, being remarkable the finding of 5–9 different MLTs in each of the most exhaustively sampled farms (n: 5–13 specimens). The S1 Table provides details on the allelic profile of each isolate used in this study and includes data related to its origin. The HGDI value of the 12-satellite typing method was 0.987 (95% CI, 0.982–0.992). The figure was similar if only the four most discriminatory markers (ML2, TP14, GP60 and Cgd3_3850) were included in the multilocus analysis (HGDI: 0.969; 95% CI, 0.960–0.977), although the number of MLTs was dramatically reduced (n: 44).

**Table 3:** Multilocus subtypes (MLTs) of *C*. *parvum* isolates from calves based on the combination of alleles at GP60 and eleven VNTR loci.

MLT	Allele at locus^a^												No. Isolates (n = 102)^b^	No. Farms (n = 29)
	MSB	CP47	Cgd1_3670	Cgd6_3940	ML1	Cgd6_5400	5B12	MSC6-7	Cgd3_3850	TP14	ML2	GP60		
1	3	1	1	3	2	1	1	1	4	1	5	3	1	1
2	3	1	2	1	2	1	3	1	4	1	5	3	1	1
3	3	1	2	2	2	1	3	1	4	1	5	3	2	1
4	3	1	2	2	2	1	3	1	4	2	5	3	1	1
5	3	1	2	2	2	1	3	1	4	2	7	3	1	1
6	3	1	2	2	2	1	3	1	4	2	9	3	1	1
7	3	1	2	2	2	1	3	2	5	1	6	3	1	1
8	3	1	2	2	2	1	4	2	5	1	6	3	1	1
9	3	1	2	3	1	1	3	1	4	1	6	3	1	1
10	3	1	2	3	1	1	3	1	4	2	6	3	1	1
11	3	1	2	3	1	1	3	1	4	3	5	3	3	1
12	3	1	2	3	1	1	3	1	4	3	7	3	2	1
13	3	1	2	3	2	1	1	1	1	2	5	5	1	1
14	3	1	2	3	2	1	1	1	4	1	2	3	1	1
15	3	1	2	3	2	1	1	1	4	2	5	3	1	1
16	3	1	2	3	2	1	1	1	4	2	6	5	1	1
17	3	1	2	3	2	1	2	1	4	3	7	5	1	1
18	3	1	2	3	2	1	2	2	4	2	6	3	1	1
19	3	1	2	3	2	1	3	1	1	2	5	5	2	1
20	3	1	2	3	2	1	3	1	1	2	7	3	1	1
21	3	1	2	3	2	1	3	1	2	3	5	3	1	1
22	3	1	2	3	2	1	3	1	3	2	4	3	1	1
23	3	1	2	3	2	1	3	1	3	2	6	3	1	1
24	3	1	2	3	2	1	3	1	4	1	3	3	1	1
25	3	1	2	3	2	1	3	1	4	1	4	3	2	2
26	3	1	2	3	2	1	3	1	4	1	4	5	1	1
27	3	1	2	3	2	1	3	1	4	1	5	3	4	2
28	3	1	2	3	2	1	3	1	4	1	6	3	2	2
29	3	1	2	3	2	1	3	1	4	1	6	5	1	1
30	3	1	2	3	2	1	3	1	4	1	7	5	4	2
31	3	1	2	3	2	1	3	1	4	2	3	3	5	2
32	3	1	2	3	2	1	3	1	4	2	4	3	3	3
33	3	1	2	3	2	1	3	1	4	2	5	3	3	3
34	3	1	2	3	2	1	3	1	4	2	5	5	2	2
35	3	1	2	3	2	1	3	1	4	2	6	3	1	1
36	3	1	2	3	2	1	3	1	4	2	6	5	1	1
37	3	1	2	3	2	1	3	1	4	2	8	3	5	2
38	3	1	2	3	2	1	3	1	4	3	3	3	3	2
39	3	1	2	3	2	1	3	1	4	3	3	5	2	1
40	3	1	2	3	2	1	3	1	4	3	5	3	3	2
41	3	1	2	3	2	1	3	1	4	3	5	5	3	2
42	3	1	2	3	2	1	3	1	4	3	6	5	1	1
43	3	1	2	3	2	1	3	1	4	3	7	3	6	1
44	3	1	2	3	2	1	3	1	4	3	7	5	1	1
45	3	1	2	3	2	1	3	1	5	1	6	3	2	2
46	3	1	2	3	2	1	3	1	6	2	4	2	2	1
47	3	1	2	3	2	1	3	2	4	1	14	3	1	1
48	3	1	2	3	2	1	3	2	5	1	6	3	5	2
49	3	1	2	3	2	1	3	2	5	1	6	5	1	1
50	3	1	2	3	2	1	3	2	5	3	6	5	1	1
51	3	1	2	3	2	1	4	1	4	2	3	3	1	1
52	3	1	2	3	2	1	4	1	4	2	9	3	1	1
53	3	1	2	3	2	1	4	2	4	2	3	3	1	1
54	3	1	2	3	2	2	2	1	4	1	9	3	1	1
55	3	1	2	3	2	2	3	1	4	1	3	3	1	1
56	3	1	2	3	2	2	3	1	4	1	6	3	3	2
57	3	1	2	3	2	2	3	1	4	1	9	3	4	2
58	3	1	2	3	2	2	3	1	4	2	4	3	1	1
59	3	1	2	3	2	2	3	1	4	3	5	5	1	1
60	3	1	2	3	2	2	4	1	4	1	9	3	1	1
61	3	1	2	3	2	3	3	1	1	3	5	3	1	1
62	3	1	2	3	2	3	3	2	4	1	7	3	1	1
63	3	1	2	3	4	1	3	1	4	2	13	3	1	1
64	3	1	2	3	4	1	3	1	4	3	13	3	1	1
65	3	1	3	3	2	1	3	2	5	1	6	3	1	1
66	3	1	4	3	1	1	3	1	4	2	5	6	1	1
67	3	1	4	3	1	1	3	1	4	3	5	6	1	1
68	3	1	4	3	2	1	3	1	5	2	6	3	1	1
69	3	1	4	3	2	1	3	1	5	2	14	1	1	1
70	3	2	2	3	2	1	2	1	4	1	5	4	2	1

^a^ The number allocation for alleles is indicated in Table 2

^b^ Only those isolates typable at all loci were used for multilocus analyses. Samples with mixed infections at a single locus were allocated to the corresponding MLT.

### Population analysis

**Fig 2:**
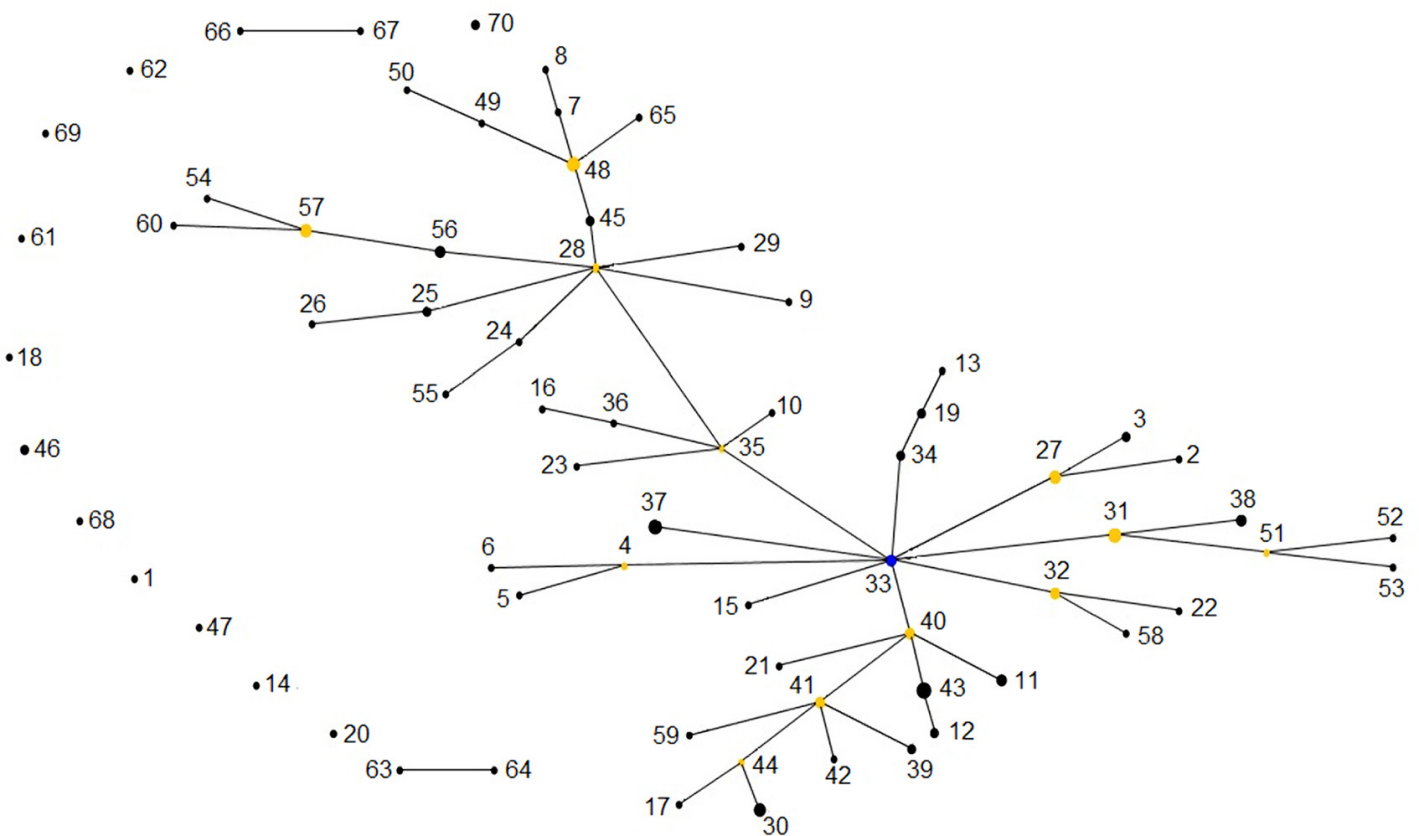
Single-locus variant eBURST network for 70 multilocus subtypes (MLTs) identified among *Cryptosporidium parvum* isolates from calves. Dots represent MLTs, with diameters proportional to numbers of isolates. Single locus variants are joined by lines. Distance between dots is random and does not provide additional information. The allelic profile of each MLT is indicated in Table 3.

**Fig 3:**
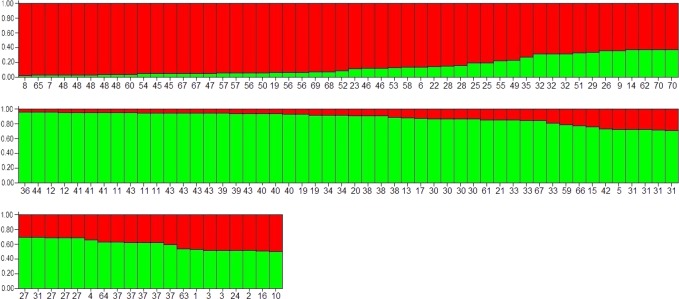
Population sub-structure based on Bayesian clustering (*K* = 2) for 102 *Cryptosporidium parvum* isolates from calves. Each bar represents an isolate, the colors within the bar reflect the percent assignment (shown on the y-axis) of that specimen to one of two genetic clusters (red or green, respectively). The MLT number for each isolate is shown on the x-axis. Analyses were conducted on allelic data at all VNTR loci and the GP60 marker. The allelic profile of each MLT is indicated in Table 3.

The BURST algorithm used to evaluate the relationships among 70 MLTs revealed the presence of two main clusters with a star-like topology, and MLTs 33 and 28 as predicted founders (bootstrap values: 81% and 75%, respectively). These founders were identified in five farms and were linked by MLT 35. Altogether, a total of 55 MLTs were linked by SLVs. The remaining 15 MLTs from twelve farms were singletons not clustered in any clonal complex, including two specimens with a biallelic profile at the TP14 locus that were allocated to linked MLTs (63–64 and 66–67) (Fig 2). Linkage analyses yielded an *I*_A_^S^ value of 0.0065 and the pairwise variance (*V*_D:_ 1.8371) was lower than the 95% critical value (*L*: 1.9427) when all specimens were analyzed as a single population (*P* > 0.05), revealing the presence of linkage equilibrium (LE) and a predominant panmictic structure within the *C*. *parvum* population in this geographical area. The LE was maintained when isolates of the same MLT were scored as a single individual (*I*_A_^S^: - 0.0071). The evolutionary relationship among specimens was inferred using STRUCTURE. An isolate was considered to belong to one of the clusters only if the probability of it belonging was higher than 0.8. Otherwise, the isolate was considered to have a mixed ancestry. The Bayesian analysis identified *K* = 2 to be the best estimation of ancestral source populations from these *C*. *parvum* isolates (Fig 3). Both clusters were not related to the geographic region or GP60 subtype and included 41 and 36 specimens, respectively. Nevertheless, *F* statistics indicated that these two sub-populations were not significantly different (*F*st: 0.01; 18.3% of *F*st values being greater that this one in a 500 permutations test in a single population). Comparison between the two groups provided Nm value of 25, a high figure as could be expected in the absence of strict reproductive isolation. Most MLTs allocated to different complexes by eBURST analyses also showed different ancestral lineages by Bayesian analysis. A total of 43 specimens from nineteen farms could not be assigned to only one cluster as they showed mixed ancestry.

## Discussion

All selected markers in the current study were in coding regions for hypothetical or known proteins or enzymes and were selected based on their variability in previous studies, except for the newly described microsatellites. In fact, most loci exhibited various degrees of polymorphism, with ML2, TP14 and GP60 being the most discriminatory and other which may be dismissed as monomorphic (MSB) or less informative (CP47, ML1 and the novel loci Cgd1_3670 and Cgd6_3940). Stuttering associated with di-nucleotide VNTR loci such as ML2 and 5B12, previously reported as a problem for sequence based approaches, was overcome in this study using CE fragment sizing [15, 19]. The CE-based tool also demonstrated an acceptable typeability, with 104/123 samples amplifying at all twelve loci. It is worth mentioning the finding of previously un-described alleles at three loci and the high variability of the novel locus Cgd3_3850, which seems a good candidate for future multilocus analyses.

The study shows the rationale for a proper choice of markers to reach a better cost-effectiveness when optimizing a multilocus approach. The 12-loci typing method provided a high discriminatory power, but the exclusion of the three less polymorphic markers (MSB, CP47, Cgd1_3670) hardly reduced the number of MLTs, and the inclusion of only four markers (ML2, TP14, GP60 and Cgd3_3850) hardly impaired the HGDI value, indicating a redundancy which has been detected in most multilocus schemes [28]. Nevertheless, comparison with a 6-loci typing approach previously used on cattle farms in northern Spain demonstrated that the current method unraveled a higher genetic diversity, with 70 MLTs identified within 102 *C*. *parvum* isolates as compared to 59 MLTs within 140 isolates [16]. The number is similar to that reported in calves in Ireland, where a total of 78 MLTs were seen among 245 *C*. *parvum* isolates using a multilocus study with seven markers [29], and much higher to that seen in calves in the UK using an 6-loci approach (23 MLTs) [30], but lower to that detected in isolates from humans and livestock in Scotland (95 MLTs), Italy (102 MLTs) or the United States (94 MLTs) using different panels of loci [2, 31, 32].

Studies to identify markers with the greatest potential for inclusion in multilocus typing have revealed that different sets of loci are required for each species of *Cryptosporidium*, and no standard panel has been universally adopted so far. Robinson and Chalmers (2012) [28] found that some of the loci used in the current study were more informative for *C*. *hominis* (CP47, MSB, MSC6-7, ML1), *C*. *parvum* (ML2) or had a similar potential for both species (TP14). The CP47 and MSC6-7 have been described among the less polymorphic loci in *C*. *parvum* from humans and cattle [32], but were among the more diverse and discriminatory in *C*. *hominis* [17, 33, 34]. Similarly, Hunter et al. [35] found that a 3-locus system (ML1, ML2 and GP60) was much more discriminatory for *C*. *parvum* than *C*. *hominis*. Comparison to a previous Spanish study with *C*. *parvum* from small-ruminant herds shows that some loci were much more discriminatory for typing isolates from lambs and goat kids (MSB, ML1), while other were more informative for isolates from calves (ML2 or 5B12), indicating that resolution is also determined by the host species [3].

Most MLTs (54/70) were unique to individual farms, which supports the usefulness of VNTR typing for tracking outbreak sources and indicates that cryptosporidiosis is an endemic condition on most cattle farms in this area. This finding is consistent with intensive farming practices typical of dairy herds sampled in this study, where animals are confined and exchange between herds is restricted. It would promote the maintenance and expansion of genetically unique strains in calving areas, limiting the opportunities for the transmission to other herds. However, our observations also revealed the presence of multiple MLTs on most farms where several samples were analyzed (90.5%), with up to 9 MLTs being found on one farm and the presence of individual specimens with mixed parasite subpopulations being reported on 11/29 farms. These findings indicate that concurrent involvement of *C*. *parvum* haplotypes is common in diarrheic outbreaks, in agreement with previous surveys in Spain and other European countries revealing a high level of mixed infections, which can reach up to 37% of fecal specimens according to some studies [2, 16, 31, 36]. In contrast, single MLTs were detected in most farms in the UK [30], and only 11 of the 205 sampled herds had calves with different MLTs in Ireland, a finding that the authors related to the geographical isolation of the island [29]. Studies using CE fragment analysis or other tools such as next-generation sequencing platforms, cloning or terminal-restriction fragment length polymorphism have showed that mixed infections are more of a rule than an exception, evidencing the limitations of conventional Sanger sequencing to resolve mixtures of templates [8–10, 37].

The significance of *Cryptosporidium* mixed populations at both intra-herd and intra-host levels have received limited attention, despite being critical issues if genetic exchange is occurring at any significant degree. Some herd management strategies such as animal movement between herds have been shown to influence allele diversity in bovine populations. A high number of allelic variants within the calf population but only one subtype within any herd was found in areas where cattle movement is restricted [38], while fewer subtypes were identified in areas with higher exchange rates between herds, although several subtypes could be present in a herd [39]. The same within-herd pattern has been reported by multilocus typing, with more mixed infections and more MLTs per herd in areas where exchange of calves between farmers and/or the introduction of new animals occurs frequently [29, 31, 40]. In this study, herd-to-herd transmission of the protozoan was limited by the above mentioned intensive husbandry practices, which strongly suggest that other factors such as intra-isolate recombination play a major role to explain the extensive genetic heterogeneity found within farms. Comparative genomic analyses of natural and laboratory-propagated isolates have revealed that *Cryptosporidium* exhibits a high rate of recombination, and it has an important role in the emergence and evolution of virulent variants of the protozoan [40–44].

The above-mentioned observations were supported by means of linkage analyses, which found evidence of linkage equilibrium and a predominantly panmictic structure, typical of populations where genetic exchange occurs at random with limited or no sub-structuring. Bayesian structure analysis detected two main ancestral clusters not related to geographic location or GP60 subtype, but sub-structuring was not supported by *F* statistics. Additionally, over 35% of isolates could not be clearly assigned and showed mixed origin. Likewise, the analysis of evolutionary descent by the algorithm eBURST detected a high rate of MLTs appearing as singletons (21.4%), a finding which indicates a high degree of genetic divergence [24]. Previous studies on the population structure of *C*. *parvum* using a variety of markers have generated different results and interpretations. A study by Mallon et al. [36] provided the first evidence of a predominantly panmictic structure, which was later reported in the United States and Ireland [29, 32]. Panmixia has been related to areas with a high rate of transmission, as it increases the odds of sexual recombination between genetically distinct isolates [45]. In northern Spain, the protozoan has been identified in over 76% of dairy cattle farms and 57% of diarrheic newborn calves, and longitudinal studies indicate that 100% of calves in some farms will shed oocysts at some point in the first 30 days of life, which indicates a high burden of infection [14, 46].

In contrast, the *C*. *parvum* population structure was found to be predominantly clonal in Italy [2], which was also seen for *C*. *parvum* IId isolates from China, Sweden and Egypt [4], while epidemic clonality was reported for *C*. *parvum* isolates from livestock in Spain [3]. Nevertheless, other studies have demonstrated the existence of a flexible reproductive strategy for *C*. *parvum* (co-occurrence of panmictic, clonal or epidemic structure), with variations in the prevailing pathways within limited geographical areas and according to host-related factors [45]. Evidence of these variations have been found in Scotland, where *C*. *parvum* has an epidemic structure in humans but panmictic in cattle [36], or a panmictic structure in Dumfriesshire and Aberdeenshire, but epidemic clonality in Orkney and Thurso [31]. The Italian study by Drumo et al. [2] found that sexual recombination was not frequent enough to break the prevalent pattern of clonality, although the low indexes of association and the high level of diversity suggested that some genetic exchange may occur. Similarly, the Spanish study by Quílez et al. [3] showed that linkage disequilibrium observed among and within *C*. *parvum* subpopulations from calves and lambs/goat kids could arise from the clonal expansion of one or more MLTs to produce epidemic clones, although evidence of genetic flow between the two subpopulations was also detected. It is worthy to note that the latter study was conducted in cattle farms from a much more extensive area in northern Spain, which support the presence of differences in the population structure at a local geographical level in this area of the country.

In conclusion, this study provides comparative data on the discriminatory power of different markers as candidates for a standardized fragment typing scheme of *C*. *parvum* from calves. The multilocus approach used was overall informative to reveal the uniqueness of most MLTs, and detected a high rate of mixed infections at both intra-herd and intra-host levels, which could be a consequence of frequent meiotic recombination among different haplotypes rather than co-infections due to animal exchange between herds. Data from this study also support a predominantly panmictic structure of *C*. *parvum* in the cattle population in this discrete geographical area.

## Supporting Information

S1 Table Allelic profile for *C*. *parvum* isolates used in this study. Samples exhibiting a mixed infection at a single locus were allocated to the corresponding MLT.
(XLSX)
